# Research Advances on Matrine

**DOI:** 10.3389/fchem.2022.867318

**Published:** 2022-04-01

**Authors:** Xiao-Ying Sun, Li-Yi Jia, Zheng Rong, Xin Zhou, Lu-Qi Cao, Ai-Hong Li, Meng Guo, Jie Jin, Yin-Di Wang, Ling Huang, Yi-Heng Li, Zhong-Jing He, Long Li, Rui-Kang Ma, Yi-Fan Lv, Ke-Ke Shao, Juan Zhang, Hui-Ling Cao

**Affiliations:** ^1^ College of Pharmacy, Shaanxi University of Chinese Medicine, Xianyang, China; ^2^ Xi’an Key Laboratory of Basic and Translation of Cardiovascular Metabolic Disease, Shaanxi Key Laboratory of Ischemic Cardiovascular Disease, Institute of Basic and Translational Medicine, Xi’an Medical University, Xi’an, China; ^3^ Shaanxi Key Laboratory of Chinese Herb and Natural Drug Development, Medicine Research Institute, Shaanxi Pharmaceutical Holding Group Co., LTD, Xi’an, China; ^4^ College of Life Sciences, Northwest University, Xi’an, China

**Keywords:** matrine, extraction, synthesis, derivatization, molecular mechanism, pharmacological effects

## Abstract

Matrine is an alkaloid extracted from traditional Chinese herbs including *Sophora flavescentis*, *Sophora alopecuroides*, *Sophora* root, etc. It has the dual advantages of traditional Chinese herbs and chemotherapy drugs. It exhibits distinct benefits in preventing and improving chronic diseases such as cardiovascular disease and tumors. The review introduced recent research progresses on extraction, synthesis and derivatization of Matrine. The summary focused on the latest research advances of Matrine on anti-atherosclerosis, anti-hypertension, anti-ischemia reperfusion injury, anti-arrhythmia, anti-diabetic cardiovascular complications, anti-tumor, anti-inflammatory, anti-bacterium, anti-virus, which would provide new core structures and new insights for new drug development in related fields.

## Introduction

Matrine is an alkaloid extracted and isolated from the root bark of *Sophora flavescens* by Japanese researcher Nagai (Bohlmann et al., 2010). Later, Matrine was also found in *Sophora flavescens, Sophora alopecuroides*, mountain bean root, and other leguminous *Sophora* plants (Zhang and Shen, 2018a). Matrine is a tetracyclic quinolizidine alkaloid with the chemical formula C_15_H_24_N_2_O and a molecular weight of 248.36. Matrine exists in two states of matter: solid and liquid (Zhao et al., 2015a). α-Matrine is an acicular or columnar crystal with a melting point of 76°C, β-Matrine is an orthorhombic crystal with a melting point of 87°C, δ-Matrine is a columnar crystal with a melting point of 84°C, γ-Matrine is a liquid with a boiling point of 223°C (Feng, 2005). The most common is α-Matrine, which is soluble in water, methanol, ethyl alcohol, trichloromethane, methylbenzene and is slightly soluble in petroleum ether. Two quinazine rings bind Matrine together. The molecule contains four six-membered rings. The six-membered rings are isomers of the chair and boat conformations. The chair conformation of Matrine is determined to be the least energetic and most stable of the eight conformational isomers (Fu, 2017). Matrine contains four chiral centers in the 5S, 6S, 7R, and 11R configurations (Zhang et al., 2019). Its molecular structure consists of two saturated tertiary amines and 2 N atoms, each of which includes a pair of unpaired electrons. It is alkaline due to its attraction to protons. It has an n-1 molecular structure. Oxymatrine is generated after oxidation, which can be reduced to Matrine using a reducing agent. Lactam’s system can be saponified to yield Matric Acid or its derivatives, and Matric Acid can be dehydrated and condensed to yield Matrine.

Matrine has the dual advantages of both traditional Chinese herbs and chemotherapeutic agents. On the one hand, Matrine comes from traditional Chinese herbs such as *Sophora flavescens, Sophora alopecuroides*, and mountain bean root. After thousands of years of clinical practices, it has the advantages of definite pharmacological effects, mild efficacy, and high safety of traditional Chinese herbs. On the other hand, Matrine, as a monomer, has the advantage of known definite chemical structure to facilitate to new drug development. Matrine has a wide range of pharmacological effects, such as cardiovascular protection (Liu and Guo, 2011), anti-tumor (Chen et al., 2022), anti-inflammatory (Huang et al., 2014), immunosuppression (Kan et al., 2013), etc. Matrine has unique advantages in the treatment of various chronic diseases and widely used to treat viral hepatitis, liver fibrosis, arrhythmia, and autoimmune diseases. Due to the large dosage and low pharmacological activity of Matrine, its clinical application is limited. The researchers modified and optimized the structure of Matrine to obtain new derivatives with high efficiency and low toxicity (Huang and Wang, 2016). The review introduced recent research progresses of Matrine on extraction, synthesis, and derivatization. It focused on the latest research advances of Matrine on anti-atherosclerosis, anti-hypertension, anti-ischemia-reperfusion injury, anti-arrhythmic, anti-diabetic cardiovascular complications, anti-tumor, anti-inflammatory, anti-bacterial, anti-viral, which would provide new core structures and new insights for new drug development in related fields.

## Extraction and Synthesis of Matrine

### Extraction of Matrine

The primary source of Matrine is extraced from natural plants. The common procedures are solvent extraction, ultrasonic aided extraction, and microwave-assisted extraction. Solvent extraction is appropriate for industrial manufacturing, while ultrasonic-assisted and microwave-assisted extraction are suitable for laboratory preparation. The solvent extraction method adopts the principle of similarity and compatibility (Han et al., 2018). Guo et al. (Guo et al., 2014) used the water decocting method to screen the optimum extraction conditions of Matrine: the ratio of material to liquid (g/ml) 1:8, water extraction 3 times, 2 h each time, the concentration of 1.0 g/ml, after alcohol precipitation, adjust to pH 9–11, trichloromethane extraction 3 times, matrine yielded 0.21 mg per 100 g raw materials, as shown in Table 1. Zhang et al. (Cui et al., 2007) adopted the acid water reflux method, took crude extract recovery rate as the index, and adopted the orthogonal experimental design to optimize extraction conditions. The optimal condition was as follows: 60 mesh *Sophora flavescens* powder, 0.3% HCl as solvent, 1:12 solid-liquid ratio (g/ml), reflux for 3 times, matrine yielded 0.31 mg per 100 g raw materials, as shown in Table 1. Li et al. (Li et al., 2015) optimized the best percolation extraction process of Matrine by using four-factor and three-level orthogonal experimental design. The optimal condition was as follows: coarse powder, 65% ethanol as solvent, 1:6 solid-liquid ratio (g/ml), 24 h soaking, 4 ml/min percolation speed, matrine yielded 0.15 mg per 100 g raw materials, as shown in Table 1. Solvent extraction has the characteristics of simple operation, low cost, and high extraction efficiency, which is a traditional extraction method in industrial production.

**TABLE 1:** The extraction methods, conditions and yield of Matrine.

Solvent extraction	Liquid to material ratio (g/ml)	Solvent	Extraction times	Extraction speed (ml/min)	Extraction time (min)	Extraction temperature (°C)	Extraction frequency (kHz)or power (W)	Matrine yield (mg/100 g)	Refrences
Percolation	1:6	65% Ethanol		4	-		-	0.145	Li et al. (2015)
Decoction	1:8	Pure water	3	-	120	-	-	0.21	Guo et al. (2014)
acid reflux method	1:12	0.3%Acetic acid	3	-	-		-	0.31	Cui et al. (2007)
Ultrasonic extraction	1:10	Pure water	-	-	45	80	-	0.46	Chen et al. (2017)
Optimizing Ultrasound	1:40	60% ethanol	-	-	30	50	35 kHz	0.34	Fu et al. (2015)
Microwave extraction	1:40	80% ethanol	-	-	20	75	500 W	0.48	Wang et al. (2011)

The ultrasonic extraction method is to utilize the cavitation effects, mechanical effects, and thermal effects of ultrasonic wave to disintegrate the plant cell wall, promote the outward diffusion and dissolution of intracellular active ingredients under the high-frequency vibration of ultrasonic wave, to enhance extraction efficiency (Wan et al., 2008). Chen et al. (Chen et al., 2017) found the optimal condition for ultrasonic extraction method was as follows: pure water as solvent, 1:10 solid-liquid ratio (g/ml), 45 min ultrasonic treatment, 80°C extraction temperature, matrine yielded 0.46 mg per 100 g raw materials, as shown in Table 1. Fu et al. (Fu et al., 2015) optimized the ultrasonic extraction conditions by orthogonal test to obtain low extraction temperature as follows: 1500 W ultrasonic power, 35 kHz ultrasonic frequency, 60% ethanol as solvent, 1:40 solid-liquid ratio (g/ml), 20 min soaking, 50°C extraction temperature, 32 min extraction time, matrine yielded 0.34 mg per 100 g raw materials, as shown in Table 1. The ultrasonic extraction method has a high extraction efficiency. Still, it requires more control conditions, a more complex operation, a higher cost, and fewer single extractions, making it suited for laboratory preparation.

The microwave-assisted extraction method is to use the intense penetration and high heat energy of microwave to rupture the plant cell wall and accelerate the dissolution of active ingredients. Wang et al. (Wang et al., 2011) obtained the best microwave extraction conditions by single factor orthogonal test as follows: 500 W microwave power, 80% ethanol as solvent, 1:40 liquid-material ratio (g/ml), 75°C extraction temperature, 20 min extraction time, matrine yielded 0.48 mg per 100 g raw materials, as shown in Table 1. Microwave-assisted extraction has high efficiency, but the operation is complex and suitable for laboratory preparation.

### Complete Synthesis of Matrine

**FIGURE 1:**
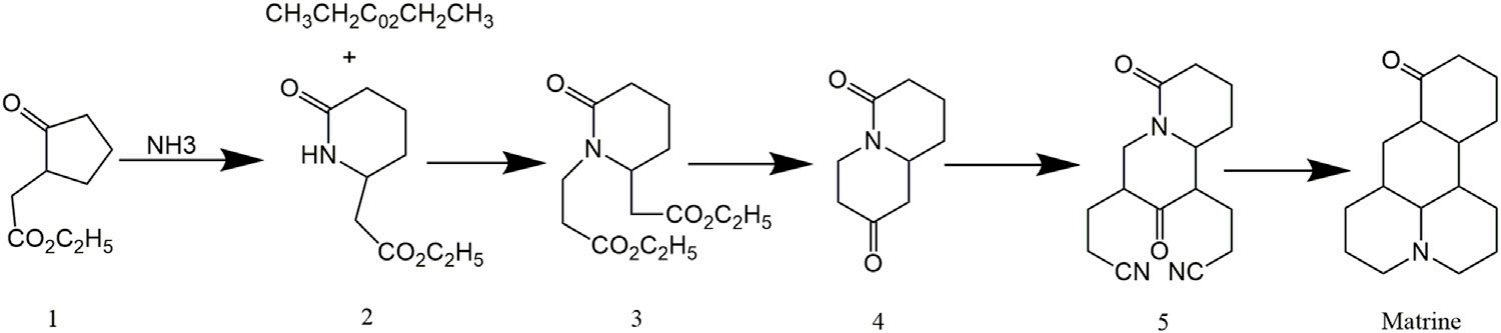
Synthetic route of Matrine by Mandell et al. (1965).

Many researchers focused on the synthesis pathway of Matrine since its discovery. There were three classical total synthesis methods of Matrine with high yield. For the first time, Leon et al. (Mandell et al., 1965) described a method for the complete synthesis of Matrine. The Dickmann and the stork enamine were used to yield Matrine from cyclopentanone 2-ethyl acetate (Figure 1-1). The overall yield of Matrine was approximately 12% with high yield, short synthetic process and convenient perform, as shown in Figure 1.

**FIGURE 2:**
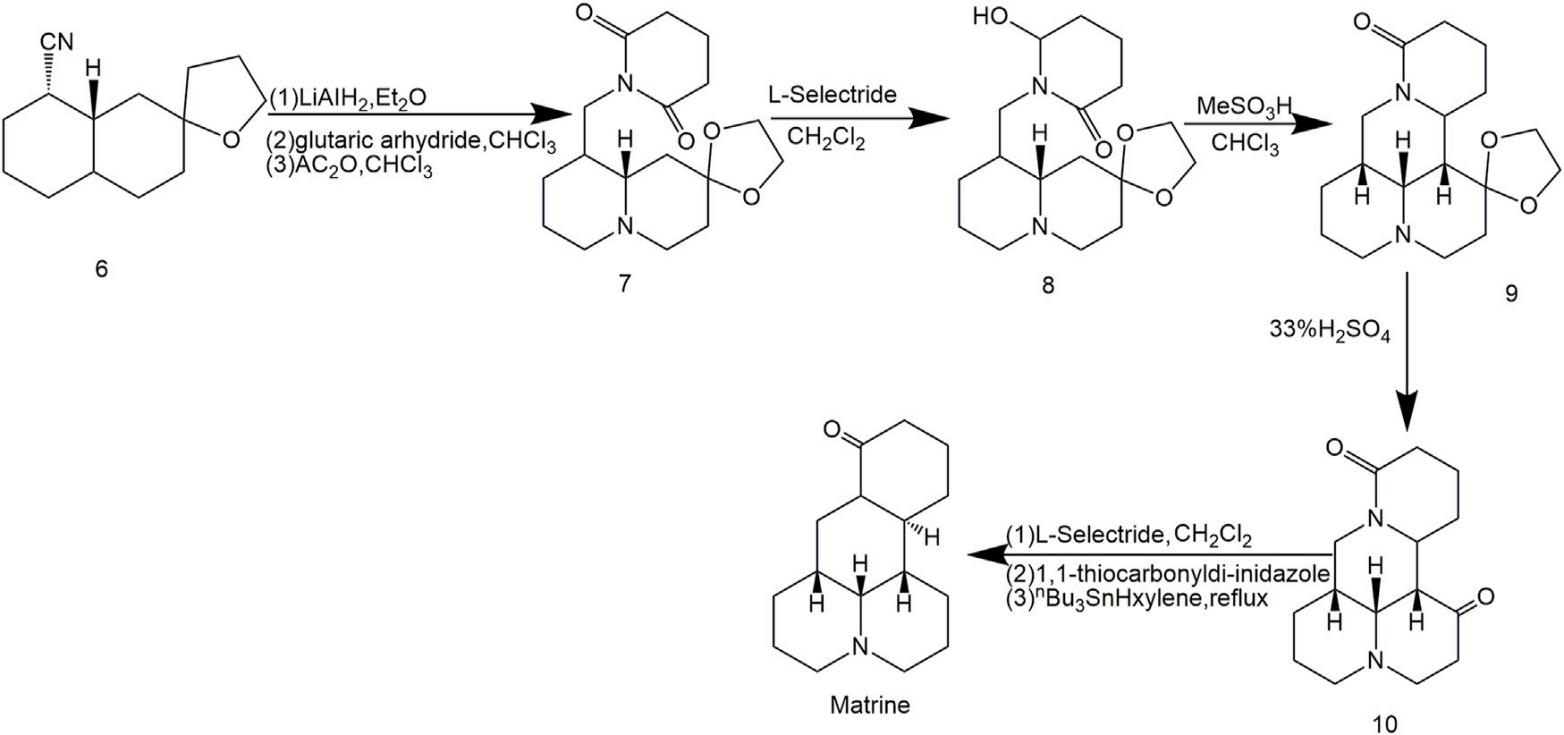
Synthetic route of Matrine by Chen et al. (1986).

**FIGURE 3:**
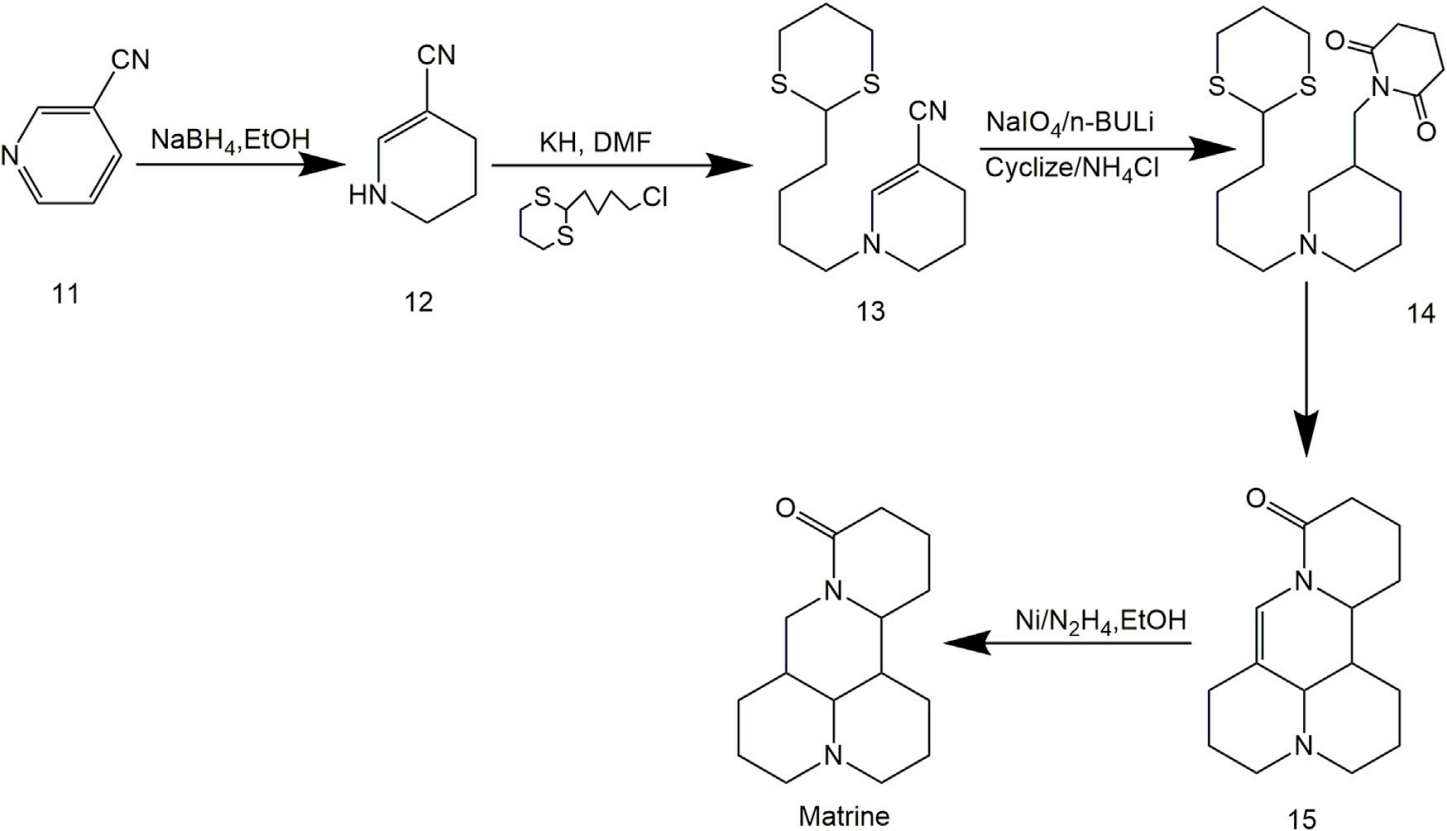
Synthetic route of Matrine by Fleming et al. (1997).

Chen et al. (Chen et al., 1986) obtained Matrine from 4-cyanoquinolisidine acetal (Figures 2-6) by selective reduction and deacetal reaction. The synthesis technique reached a new milestone for Matrine synthesis and total yield rate of Matrine jumped to 23%, as shown in Figure 2.

By intramolecular addition cyclization, Fleming et al. (Fleming et al., 1997) synthesized Matrine from unsaturated nitrile, which provided an ideal synthetic route of Matrine with fewer reaction steps, simple operations and easy-handling products, as shown in Figure 3.

## Pharmacological Effects of Matrine

### Cardiovascular and Cerebrovascular Protection Activity

Cardiovascular and cerebrovascular diseases as global people’s leading cause, its new drug development is indispensable. Matrine, as a Chinese herb monomer with multiple activities, has unique advantages in preventing and improving atherosclerosis, hypertension, ischemia-reperfusion injury, arrhythmia, and diabetic cardiovascular complications (Liu and Guo, 2011).

1) Anti-atherosclerosis activity

AS (Atherosclerosis) is the primary pathological basis of cardiovascular and cerebrovascular diseases such as hypertension, coronary heart disease, stroke, cerebral infarction and diabetic cardiovascular complications. Its molecular pathogenesis involves in inflammation, endothelial injury, immune dysfunction, lipid metabolism disorders and foam cell formation (Xu et al., 2007). The foam cells were formed by the excessive lipid phagocytosis and cholesterol accumulation by macrophages (Tsai et al., 2010). Matrine could reduce the level of LDL-C (low-density lipoprotein cholesterol) and TG (triglyceride) in the blood of hypercholesterolemic mice, hyperlipidemic rats, and pigeon models to prevent AS (Lu and Zhu, 2003). ABCA1(ATP-binding cassette transporterA1) can regulate the RCT (reverse cholesterol transport). Its dysfunction can lead to the excessive cholesterol accumulation in macrophages to form foam cells, then infiltrate blood vessel walls, and promote the occurrence and development of AS (Li et al., 2013). Matrine could upregulate the ABCA1 expression to promote RCT, reduce the cholesterol accumulation in foam cells, and improve AS (Jiang et al., 2007).

Excessive accumulation of cytotoxic free cholesterol in foam cells induces cell apoptosis, and then, lipids and cell debris can infiltrate the inner membrane of vascular walls to form AS plaques. Subsequently, macrophages release inflammatory factors, such as TNF-α (tumor necrosis factor-α), IL-6 (interleukin-6), etc, which promotes the proliferation and migration of macrophages and VSMCs (vascular smooth muscle cells) to accelerate the AS progression (Moriya, 2018). Matrine could suppress the cell inflammatory response, inhibit the proliferation and apoptosis of VSMCs to improve AS by inhibiting JAK/STAT3 (Janus kinase1/activation of signal transduction and activator of transcription3) signaling pathway (Lu et al., 2018). Additionally, Matrine could also downregulate the overexpression of VCAM-1 (vascular cell adhesion molecule-1) and ICAM-1 (intercellular adhesion molecule-1) induced by TNF-α to decrease cell adhesion to improve AS by downregulating NF-κB (nuclear factor kappa-B) and MAPK (mitogen-activated protein kinase) signaling pathways (Liu et al., 2016).

2) Anti-hypertension activity

Hypertension is an independent risk factor for cardiovascular disease. Systolic blood pressure increases every 20 mmHg, diastolic blood pressure increases by 10 mmHg, and cardiovascular risk doubles (China National Center for Cardiocascular Disease, 2020). Therefore, controlling blood pressure can significantly reduce morbidity and mortality of cardiovascular diseases. Hypertension pathogenesis involves in sympathetic nerve excitation, renin-angiotensin-aldosterone system, Ca^2+^ release, vascular remodeling and so on (He et al., 1999; Zheng et al., 2009; Wei, 2016; Li et al., 2020a; Ge and Xu, 2020; Sun et al., 2021).

Matrine could inhibit α-Adrenoceptor activation to interfere with intracellular Ca^2+^ release and extracellular Ca^2+^ influx, thereby reducing blood pressure induced by phenylephrine (Zheng et al., 2009). Abnormal proliferation of pulmonary artery smooth muscle cells is the key cause to induce pulmonary hypertension. Matrine could inhibit the proliferation of human pulmonary artery smooth muscle cells, block cell cycle from G0/G1 to S phase, promote cell apoptosis, therefore, improving pulmonary hypertension (Ge and Xu, 2020). Inflammatory cytokines such as IL-6 and TGF-β1 (transforming growth factor-β1) play an essential role in the occurrence and development of pulmonary hypertension. Inflammatory cytokines could infiltrate pulmonary artery vessels, make pulmonary artery smooth muscle cells proliferate abnormally, and induce pulmonary vascular remodeling. Matrine could downregulate TNF-α and IL-1β expression by NF-κB signaling pathway to prevent vascular injury and protect pulmonary vessels, thereby, improving pulmonary hypertension in rats. In addition, Matrine could improve pathological changes of the lung in pulmonary hypertension model mice by inhibiting oxidative stress (Li et al., 2020a). Cardiovascular remodeling and target organ injury are closely related to the occurrence and development of hypertension. Hypertension treatment can effectively reduce blood pressure, reverse cardiovascular remodeling, prevent myocardial fibrosis, and protect target organ injury. Matrine could improve vascular remodeling by inhibiting the abnormal proliferation of VSMCs induced by AngII (angiotensinII) (He et al., 1999). RhoA (Ras homologous gene family proteinA)/Rock1(Rho related coiled-coil forming protein kinase1) signaling pathway is closely related to myocardial fibrosis. Matrine could inhibit the activation of RhoA/Rock1 signaling pathway, reduce myocardial fibrosis, prevent ventricular remodeling, and improve cardiac function in rats with heart failure (Sun et al., 2021). Left ventricular hypertrophy is a complication of hypertension. Matrine could up-regulate the expression of ciliary ganglion neurotrophic factor, downregulate the expression of IGF-1 (insulin-like growth factor1), TGF-β1, ICAM-1 and macrophage inflammatory protein 2 by inhibiting NF-κB signaling pathway to improve isoproterenol-induced left ventricular hypertrophy (Wei, 2016).

3) Anti-ischemia reperfusion injury activity

I/R (ischemia reperfusion injury) refers to that the ischemic injury of tissues and organs aggravates by blood reperfusion after ischemia, which involves in oxidative stress and inflammatory response. Matrine could significantly downregulate MDA (malondialdehyde), upregulate SOD (superoxide dismutase) and GSH (glutathione peroxidase) to inhibit oxidative stress in rats after cerebral I/R, significantly downregulate caspase-3 and the ratio of Bax/Bcl-2 to inhibit apoptosis, and therefore protect nerves cells (Zhao et al., 2015b). Matrine could also inhibit oxidative stress to alleviate acute liver injury after hepatic I/R by inhibiting NF-κB pathway (Zhang et al., 2011a).

Hsp70 (heat shock protein 70) is a cardiac protective molecule that can inhibit cardiomyocyte apoptosis after myocardial I/R or hypoxia/reperfusion injury. Matrine could up-regulate Hsp70 expression by activating JAK2/STAT3 pathway to protect cardiomyocyte after hypoxia/reperfusion injury (Guo et al., 2018). AMPK (AMP-dependent protein kinase) and SIRT3 (sirtuin3) are classical anti-apoptotic pathways involved in the occurrence and development of many diseases. Matrine could activate the AMPK/SIRT3 signaling pathway, inhibit cardiomyocyte apoptosis induced by I/R to protect cardiomyocyte (Lu et al., 2020). Matrine could down-regulate the level of Akt phosphorylation at Thr495 and up-regulate the expression of eNOS (endogenous nitric oxide synthase), downregulate the expression of DDAH2 (dimethylarginine dimethylaminohydrolase2) and up-regulate phosphorylation level of GSK-3β (glycogen synthase kinase3β), reduce subendocardial necrosis, relieve inflammatory cell infiltration and interstitial edema, and improve the acute myocardial I/R injury induced by super dose isoproterenol in rats (Li et al., 2012a).

4) Anti-arrhythmia activity

Arrhythmia is a heart disease characterized by abnormal heartbeat frequency and rhythm. Electrophysiology is characterized by changes in the origin, conduction velocity, or activation order of electrical activities in the myocardium. The arrhythmia pathogenesis includes spontaneous depolarization, abnormal electrical triggering and reentry, abnormal action potential and so on (Zhang et al., 2020a), which involves in ion channels on cardiomyocytes, such as sodium channel, calcium channel, potassium channel, etc (Bers et al., 2003; Antzelevitch and Belardinelli, 2006; Jost et al., 2009; Sun and Wang, 2010; Varró et al., 2010; Passini et al., 2014; Wu et al., 2020). Matrine could block the increase of L-type Ca^2+^ current and Ca^2+^ transient, up-regulate L-type calcium channel current, activate M3 receptor, up-regulate the expression of IkM3 (delayed rectifier potassium current), prolong the time course of the action potential, restore the transient outward potassium current and inward rectifier potassium current of rat ventricular myocytes after myocardial infarction, and reduce Ca^2+^ in cardiomyocytes, therefore, improve ouabain induced action potential prolongation and arrhythmia in rats, and improve arrhythmia caused by coronary artery ligation or myocardial infarction in rats (Zhou et al., 2014a). Matrine could inhibit cardiomyocyte apoptosis to prevent heart failure in rats by inhibiting the β3-Ar pathway, (Yu et al., 2014).

Cardiac fibrosis promotes atrial fibrillation by disrupting the continuity of fiber bundles and causing abnormal local electrical conduction (Research, 2009; Yue et al., 2010). Matrine could improve myocardial fibrosis in mice induced by aortic ligation or injection of isoproterenol by up-regulating ribosomal protein S5 and inhibiting p38 activation, therefore, reduce the susceptibility to atrial fibrillation after myocardial infarction, and shorten the onset time of atrial fibrillation (Antzelevitch and Belardinelli, 2006).

5) Anti-diabetic cardiovascular complications activity

DM (diabetes mellitus), a complex and multifactorial metabolic disease, is characterized by with elevated blood glucose levels induced insulin resistance and β-Cell failure. T2DM (type 2 diabetes mellitus) accounts for majority of DM patients. The cardiovascular risk in DM is 2–4 times higher than that in normal subjects. A high glucose environment in DM patients will produce AGEs (advanced glycation end products) by glycosylated modification of macromolecules such as protein, lipid, and nucleic acid. It is the main pathogenic pathway and the primary source of glucotoxicity in DM patients to induce intracellular pathological changes induced by AGEs binding with its receptor RAGE (receptor for advanced glycation end products). AGEs could transform HCSMCs (human coronary artery smooth muscle cells) from contractile to the synthetic phenotype to lose their contractility and up-regulate ECM (extra cellular matrix) expression to induce AS to induce diabetic cardiovascular complications. Matrine could down-regulate RyR2 expression, inhibit calcium overload caused by endoplasmic reticulum leakage to protect mitochondrial function, reduce age-induced heart injury and inhibit cardiomyocyte apoptosis (Wang et al., 2019a). Matrine could up-regulate poldip2 (polymerase δ Interacting protein2) expression, down-regulate Akt and mTOR phosphorylation, down-regulate phosphorylation level of downstream effector translation regulator P70S6K (70 kDa ribosomal S6 kinase), inhibit ECM protein expression, therefore, inhibit age-induced phenotypic transformation and fibrosis of HCMC (Ma et al., 2019).

Hyperglycemia can promote myocardial fibrosis and scar formation, and lead to changes in myocardial structure. Matrine could downregulate expression of TNF-α, IL-6 in serum and cardiomyocytes of rats to alleviate metabolic disturbance syndrome and vascular injury in streptozotocin-induced diabetic cardiomyopathy rats. Matrine could downregulate TGF-β1 expression and phosphorylation level of cardiomyocyte PERK (protein kinase RNA like endoplasmic reticulum kinase) to reduce cardiomyocyte apoptosis (Hou et al., 2019). Matrine could down-regulate TGF-β1 and Smad expression to improve the heart compliance and left ventricular function of diabetic myocardial fibrosis mice (Zhang et al., 2018a). Matrine could downregulate NFAT (nuclear factor of activated T-cell) signaling pathway and ECM gene expression to improve diabetic myocardial fibrosis and to protect cardiac function (Liu et al., 2017).

Oxidative stress is closely related to cardiovascular complications in DM. Under high glucose stimulation, ROS (reactive oxygen species) will release significantly, triggering apoptosis cascade reactions and inducing cardiomyocyte apoptosis in diabetic cardiomyopathy. Matrine could inhibit the activation of the ROS/TLR-4 signaling pathway, reduce the ROS in cells, inhibit the cardiomyocyte apoptosis, and improve heart function in diabetic cardiomyopathy mice (Liu et al., 2015). Matrine could dose-dependently reduce cellular ROS, down-regulate the expression of NLRP3 (NLR family protein3) inflammatory bodies, inhibit the secretion of inflammatory factors, reduce cell apoptosis and inhibit age-induced arterial cell injury (Zhang et al., 2018b).

Matrine could inhibit the release of inflammatory factors, regulate VEGF (vascular endothelial growth factor) and angiopoietin-1, inhibit the proliferation of retinal microvascular endothelial cells, and reduce diabetic retinopathy (Zhang, 2019). Matrine could downregulate TNF-α and IL-6 expression in serum, reduce the excretion rate of urinary microprotein caused by an inflammatory reaction, increase insulin sensitivity and assist treatment of T2DM nephropathy (Lu et al., 2015).

### Anti-Cancer Activity

The malignant tumor is the second leading death cause worldwide and chemotherapy is the major treatment way. The toxic side effects from chemotherapy seriously threaten cancer patients’ health and life quality. Matrine, a Chinese herb monomer, has the dual advantages of traditional Chinese herbs and chemotherapy drugs, which could provide a new core structure and new insights for anti-cancer new drug development. Matrine injection has been clinically used as an anti-tumor adjuvant therapy in China. Matrine could inhibit the proliferation of a variety of tumor cells, induce cell cycle arrest, promote apoptosis, inhibit metastasis and invasion, reverse multidrug resistance, reduce the toxicity of radiotherapy and chemotherapy, and show favorable anti-tumor activity (Warburg, 1956; Zhang et al., 2009a; Li et al., 2012b; Zhou et al., 2012; Shao et al., 2013; Zhou et al., 2014b; Liu, 2014; Raimondi et al., 2014; Hu et al., 2015a; Wang et al., 2015; Zhang et al., 2015; An et al., 2016; Chan et al., 2017; Wu et al., 2017; Xiao et al., 2017; Zhou, 2017; Zhuo and Ji, 2017; Pu et al., 2018; Xiao et al., 2018; Xie et al., 2018; Zhou et al., 2018; Dai et al., 2019a; Wang et al., 2019b; Chen et al., 2019; Wang et al., 2019c; Dong, 2019; Guo et al., 2019; Hao et al., 2019; Kang et al., 2019; Lin et al., 2019; Liu et al., 2019; Li et al., 2020b; Ren et al., 2020; Dai et al., 2021a).

1) Anti-lung cancer activity

The occurrence and development of lung cancer are related to the imbalance of multiple signal pathways, such as the activation of PI3K/Akt/mTOR signaling pathway (Hao et al., 2019), CCR7(chemokine receptor7) (Pu et al., 2018), EGFR (Zhuo and Ji, 2017), and the high expression of TMEM16A (transmembrane protein 16A) (Guo et al., 2019). Matrine could inhibit PI3K/Akt/mTOR signaling pathway (Hao et al., 2019), down-regulate the expression of CCR7, EGFR, TMEM16A, down-regulate the ratio of apoptosis-related protein Bcl-2/Bax, inhibit cancer cell proliferation (Xie et al., 2018), migration and invasion (Zhang et al., 2009a), inhibit angiogenesis (Zhou et al., 2012), induce apoptosis (An et al., 2016), and inhibit the xenograft growth in tumor-bearing mice model (Hao et al., 2019).

The activation of cancer-promoting factor EGFR can up-regulate the expression of IL-6, JAK1/STAT3 signaling pathway and deteriorate NSCLC (non-small cell lung cancer), which was the reason for drug resistance. Additionally, drug resistance of lung cancer cells is also related to β-catenin/survivin, Nrf2/annexina4, etc. Matrine could inhibit the growth of NSCLC cell line H1975 by inhibiting EGFR and inhibiting the activation of the IL-6/JAK1/STAT3 signaling pathway. Matrine combined with afatinib could reverse the drug resistance of afatinib resistant strain H1975 cells by inhibiting the activation of the IL-6/JAK1/STAT3 signaling pathway (Chan et al., 2017). Matrine combined with cisplatin, 5-fluorouracil, and paclitaxel could enhance its anti-proliferation effects on A549 cells (Wang et al., 2015). β-catenin pathway is a potential target pathway to improve the sensitivity to cisplatin of tumor cells. Matrine could reverse the drug resistance of cisplatin-resistant strains of A549 and H460 cells and to induce cell apoptosis of mitochondrial pathway by inhibiting β-catenin/survivin signaling pathway (Pu et al., 2018). The combination of low-dose Matrine and cisplatin could reverse the drug resistance of NSCLC cell lines A549 and DDP to cisplatin and promote cell apoptosis, which was related to the regulation of Nrf2/annexina4 (Liu et al., 2019).

2) Anti-hepatoma activity

Tumor stem cells are the basis of unlimited tumor proliferation. CD90, CD133 and EpCAM are the stem markers of liver cancer cells. Matrine could significantly inhibit the activation of Akt in hepatoma cell lines HepG2 and Huh7, inhibit the expression of stem cell genes such as CD90, CD133 and EpCAM, and then inhibit the proliferation and metastasis of hepatoma cells (Dai et al., 2019a). Matrine could inhibit cell proliferation (Zhou, 2017), metastasis (Kang et al., 2019), promote apoptosis (Zhou, 2017), and inhibit the xenograft growth of human hepatoma cell line HepG2 in tumor-bearing mice model, which was related to inhibiting ERK signaling pathway (Liu et al., 2017), inhibiting the expression of miRNA-122, livin and survivin genes (Zhou, 2017), activating cysteine protease independent pathway and promoting programmed cell death (Zhou et al., 2014b). Matrine combined with cisplatin could enhance inhibitory of cisplatin on xenograft growth of HepG2 in tumor-bearing mice model, which was related to down-regulating the expression of survivin, up-regulating the expression of caspase-3, and promoting the apoptosis of liver cancer cells (Hu et al., 2015a).

Tumor cell metastasis is the primary cause of cancer death and recurrence, closely related to the activation of EMT to loss cell adhesion. Hypoxic conditions are the microenvironment required for tumor migration and mir-199a-5p, and HIF-1 α are the critical factor of tumor metastasis (Raimondi et al., 2014). Under hypoxia, Matrine could inhibit the cell migration of human hepatoma cell lines BEL7402 and SMMC-7721, inhibit the xenograft growth of in hepatoma tumor-bearing mice model, which was attributed to up-regulating the expression of mir-199a-5p to induce HIF-1α downregulation, followed to activate EMT (Dai et al., 2021a). Matrine could significantly inhibit the migration and invasion of human hepatoma cell lines PLC/PRF/5 and MHCC97l, which was related to downregulating the expression of MMP-9 to inhibit degradation of extracellular matrix. Wang et al. (Wang et al., 2019b) found that Matrine could inhibit the cell proliferation, migration, and invasion of human hepatoma cell line SMMC-7721, which was related to the expression of Myc proto-oncogene protein, intercellular adhesion factor-1, EGFR, cysteine protease3, and MMP-2.

3) Anti-breast cancer activity

Matrine could inhibit the proliferation, migration and invasion, promote apoptosis of human breast cancer cell lines 4T1 (Dong, 2019), MCF-7 (Li et al., 2012b), BT-474 and MDA-MB-231 (Shao et al., 2013), inhibit xenograft growth in tumor-bearing mice model (Dong, 2019), which was attributed to inhibiting Wnt/β-Catenin pathway (Xiao et al., 2018), inhibiting the expression of miR-21, activating downstream phosphatase and tensin homologues, downregulating Akt phosphorylation level, activating JNK1/AP-1 signaling pathway, downregulating p53 expression (Shao et al., 2013), down-regulating VEGF expression (Xiao et al., 2018), up-regulating the ratio of apoptosis-related protein Bax/Bcl-2 (Dong, 2019).

LIN28A is a common biomarker and therapeutic target for breast cancer, while LET-7b is a tumor suppressor. LIN28A can inhibit the expression of LET-7b, which plays a key role in regulating breast cancer stem cell renewal and tumorigenesis. Matrine could inhibit the proliferation and differentiation of human breast cancer cell lines McF-7 and T47-D by down-regulating LIN28A expression and up-regulating LET-7b expression, inhibiting Wnt/β-catenin pathway, down-regulating CCND1 (cyclin-D1) expression, and inhibiting the proliferation and differentiation of human breast cancer cell lines McF-7 and T47-D (Li et al., 2020b). Endoplasmic reticulum stress leads to mitochondrial dysfunction and promotes cell apoptosis. Matrine could activate endoplasmic reticulum stress, down-regulate the expression of hexokinase2 to promote apoptosis of MCF-7 cells (Xiao et al., 2017). ITGB1 (integrin β1) is highly expressed in invasive breast cancer cells and plays a crucial role in breast cancer cell migration. Matrine could down-regulate ITGB1 and ETM and inhibit the proliferation and migration of human breast cancer cell lines MDA-MB-231 and MCF-7 (Ren et al., 2020).

ABCB1 (ATP binding cassette transporter B1) transporter is activated to promote the efflux of intracellular chemotherapeutic drugs to produce multidrug resistance. The combination of Matrine and doxorubicin could downregulate ABCB1 expression, reduce doxorubicin efflux and reverse the drug resistance of breast cancer resistant K562/ADR cells. Matrine could activate NF-κB signaling pathway to promote resistant K562/ADR cell apoptosis (Chen et al., 2019). Matrine could inhibit PI3K/Akt signaling pathway to down-regulate the expression of MDR1 (multidrug resistance1) and MRP1 (multidrug resistance-associated protein1), thereby reverse the drug resistance of MCF-7/ADR (Zhou et al., 2018).

4) Anti-leukemia activity

Tumor cells also use glycolysis to produce energy when oxygen is sufficient, that is, the Warburg effects (Warburg, 1956). Matrine could down-regulate the expression of hexokinase 2, inhibit glycolysis level, inhibit cell energy metabolism, inhibit the proliferation of human chronic myeloid leukemia cell line K562 and promote cell apoptosis (Lin et al., 2019). Matrine could induce K562 cell cycle arrest in the S phase, delay the G2/M phase, inhibit mitosis, inhibit K562 cell proliferation and induce apoptosis, which is related to the up-regulation of p27kipl protein expression (Wang et al., 2019c). The combination of NK (natural killer cells) and its receptor NKG2D helps lyse leukemia cells. NKG2D in leukemia cells is downregulated to avoid immune cell killing. Matrine could up-regulate NKG2D expression in K562 cells, activate NK cells, release pro-inflammatory factors, increase NK cytotoxicity to K562 cells and inhibit the proliferation of K562 cells (Zhang et al., 2015).

Matrine could inhibit the growth and proliferation of human acute myeloid leukemia cell lines HL-60, THP-1, and C1498, which was related to inhibiting the Akt/mTOR signaling pathway (Wu et al., 2017).

PML/RARA (promyelocytic leukemia/retinoic acid receptor A) fusion protein can block the differentiation and maturation of granulocytes, which is the main cause of APL (acute promyelocytic leukemia). ATRA (all trans-retinoic acid) is widely used to treat APL, but it is susceptible to drug resistance. Matrine could cooperate with ATRA to inhibit telomerase activity and PML/RARA fusion protein expression, promote the degradation of PML/RARA fusion protein, up-regulate the expression of phospholipid reptilase1, and thereby reverse the drug resistance of ATRA (Liu, 2014).

### Anti-Inflammatory, Anti-Bacterium and Anti-Virus Effects

1) Anti-inflammatory activity

NF-κB signaling pathway is closely related to the secretion of inflammatory factors TNF and IL. Matrine could inhibit the NF-κB pathway, down-regulating pro-inflammatory factor TNF-α and IL-1β expression, down-regulating the expression of MMP2 and MMP3, and reducing the inflammatory response synovial tissue loss of type II CIA (collagen-induced arthritis) model mice. Matrine could inhibit the NF-κB pathway, down-regulate COX-2 (cyclooxygenase2) and iNOS expression to alleviate the pain caused by inflammatory response (Pu et al., 2016). Lu et al. (Lu et al., 2019) found that Matrine could clinically improve telangiectasia and edema caused by acute inflammation, which presented similar curative effects with aspirin.

Inflammatory mediator HMGB1 (high mobility group box1) is closely related to autoimmune encephalomyelitis. HMGB1 binding with Toll2 (Toll receptor2) can activate NF-κB pathway, promoting the release of pro-inflammatory factors and aggravating the inflammatory response. Matrine could inhibit HMGB1/Toll2/NF-κB pathway, downregulating TNF-α, IL-6, and IL-1β expression, inhibit inflammatory infiltration, reduce inflammatory injury in encephalomyelitis model mice (Chu et al., 2021). Matrine could up-regulate the expression of mirRNA-9 and inhibit JNK and NF-κB pathway to improve the inflammatory injury of PC12 cells induced by lipopolysaccharide and reduce the secondary damage of spinal cord injury in mice (Jiang and Jiang, 2020).

Matrine could inhibit the NF-κB pathway, down-regulate the expression of SOCS3 (suppressor of cytokine signaling proteins 3), inhibit the release of pro-inflammatory cytokines, inhibit inflammatory factors to stimulate airway epithelial cells to improve the symptoms of allergic airway inflammation in mice (Sun et al., 2016). Matrine could promote neutrophil apoptosis and reduce lung inflammation and injury caused by cigarette smoke (Yu et al., 2019).

In clinical trials, Matrine suppositories was used to treat chronic pelvic inflammatory disease, and the relieving effects of Matrine treatment group was better than that of the control group. Serum TNF-α, IL-1β, and IL-6 in Matrine group were significantly lower than that of the control group (Liu et al., 2020). 2, 4, 6-trinitrobenzene sulfonic acid can induce colitis in mice and cause intestinal flora imbalance. Matrine could reduce the secretion of inflammatory factors, regulate intestinal flora, and alleviate colon injury in mice (Li et al., 2019). Retinal neuritis is the leading cause of visual impairment in adolescents. Matrine could down-regulate NF expression, inhibit the release of pro-inflammatory cytokines, up-regulate Bcl-2/Bax ratio, reduce inflammatory infiltration and demyelination, and reduce retinal optic nerve ganglion cell apoptosis in retinal neuritis model mice (Kang et al., 2021).

2) Anti-bacterium activity

Matrine has broad-spectrum anti-bacterial effects. It has inhibitory effects on cocci and bacilli, such as *Staphylococcus aureus* and *Escherichia coli*, and has certain inhibitory effects on fungi. The formation of bacterial biofilm can protect bacteria from drug and environmental stimuli to produce drug resistance. Matrine could increase the membrane permeability of *Staphylococcus epidermidis* to induce its death in a dose-dependent manner, the efficacy was better than ciprofloxacin and erythromycin (Ren et al., 2020). Matrine had favorable inhibitory effects on *Staphylococcus aureus* (MIC, 25 μg/ml) (Chen et al., 2019), *E. coli* (MIC, 12.5 μg/ml) (Zhu, 2014), *Bacillus subtilis* (MIC, 12.5 μg/ml) (Liu et al., 2011), *Pseudomonas aeruginosa* (MIC, 25 μg/ml) (Zhang et al., 2011b), *Candida albicans* (MIC, 25 μg/ml) (Zhang et al., 2011b). Jing et al. (Jing et al., 2019) showed that Matrine could reduce endometritis caused by phosphoteichoic acid of *Staphylococcus aureus*, which was attributed to inhibiting TLR2/NF-κB signaling pathway.

Zhang et al. (Zhang et al., 2020b) showed that Matrine had favorable inactivation effects on *Aspergillus fumigatus*, *Trichophyton mentagrophyte*, and *Cryptococcus neoformans*. Fluconazole is a commonly used drug for fungal infections. With the increase of drug use times, fungi can develop drug resistance. Matrine could inhibit the transformation of *Candida albicans* from yeast to mycelium and reverse the drug resistance of fluconazole-resistant *Candida albicans* (Shao et al., 2014). 90% of pecan trees will be infected with pecan dry rot fungi, causing substantial economic losses to farmers. Pan (Pan, 2018) found that Matrine could act on pecan dry rot fungi, which could change the permeability of fungal cell membrane, inhibit the spore germination, inhibit the mycelial growth, inhibit the fungal glycolysis pathway, enhance aerobic respiration and produce a large number of ROS to injury pecan dry rot fungi.

3) Anti-virus activity

Yang et al. (Yang et al., 2012) found that Matrine could inhibit the RNA replication of human enterovirus 71 (EV71) in rhabdomyosarcoma cells and reduce the mortality of mice under the lethal dose of the virus. Clinical trials found that the positive serum HBV DNA in patients with chronic hepatitis B was significantly turned into negative by intramuscular injection of Matrine, which greatly improved liver function without noticeable side effects (Long et al., 2004). Animal experiments confirmed that Matrine could block the adsorption of hepatitis virus, inhibit the expression and secretion of HBsAg, HBeAg, and HBV-DNA by hepatocytes to produce anti-hepatitis virus effects (Zhang and Shen, 2018b). Yang found that Matrine sodium chloride injection had an excellent clinical impact in treating new Coronavirus pneumonia (Yang et al., 2020). Peng et al. (Peng et al., 2020) explored the mechanism of Matrine in treating COVID-19 by molecular docking technology. It was indicated that Matrine could inhibit viral replication and promote apoptosis of infected cells by downregulating TNF-α and IL-6 and up-regulating the expression of caspase-3. The mixed infection of PRRSV (porcine reproductive and respiratory syndrome virus) and PCV2 (porcine circovirus type2) could result in pig death. Nasun et al. (Sun et al., 2020) found that Matrine decreased the replication of the two viruses in the mouse liver and enhanced the immune function of mice in a dose-dependent manner. Matrine could inhibit PRRSV infection in Marc-145 cells, which was related to directly inactivate PRRSV, down-regulate PRRSV protein expression (Sun et al., 2014). Matrine had favorable inhibitory effects on tobacco mosaic virus, which was superior to commercial drug ribavirin (Ni et al., 2017).

### Other Effects

1) Analgesic activity

The N atom at position one of Matrine is an influential group for its analgesic effects, and it has noticeable analgesic effects. Haiyan et al. (Haiyan et al., 2013a) found that Matrine (7.5–30 mg/kg) could obviously reduce neuropathic pain by ligating the sciatic nerve of mice. Yang et al. (Yang et al., 2016) found that Matrine could reduce visceral inflammation and pain caused by acetic acid, physical pain caused by thermal stimulation, and neuropathic pain caused by sciatic nerve ligation, which was not attributed to central analgesia induced by activating opioid receptors. Gong (Gong, 2017) found that Matrine could reduce the neuropathic pain caused by vincristine and improve the neuropathic changes of the sciatic nerve, dorsal root ganglion, and dorsal horn of the dorsal spinal cord, which was related to inhibiting the expression of the inflammatory factor TNF-α, IL-10 and IL-6 to disable RAS (renin-angiotensin system), affecting the phosphorylation of downstream key molecule RAF (rheumatoid arthritis factor), failing activation of downstream factor ERK1/2 (extracellular regulated protein kinase1/2) and blocking the transmission of pain signals. Matrine could reduce mechanical hyperalgesia and thermal hyperalgesia in a dose-dependent manner to reduce the neuropathic pain caused by chronic constriction injury (Haiyan et al., 2013b). Acetaminophen is commonly used anti-pyretic and analgesic drug with favorable analgesic effects, but it has specific liver toxicity. The combination of Matrine and acetaminophen had better analgesic effects than acetaminophen alone and could alleviate the hepatotoxic injury caused by acetaminophen (Dai et al., 2021b). ATP combined with its receptor P2X can increase the sensitivity of neurons to induce neuropathic pain (Yin et al., 2019). When the nerve is damaged, the expression of P2X2 and P2X3 is up-regulated, and the threshold of neuropathic pain is reduced. Matrine could reduce neuropathic pain in rats by downregulating the expression of P2X2 and P2X3 in neurons (Li et al., 2020c).

2) Immunosuppressive activity

Matrine has immunosuppressive effects and Matrine suppresses autoimmune response by inhibiting the immune activity of T cells, B cells and macrophages (Ji and Zhang, 2014). Wang et al. (Wang, 2018) showed that intraperitoneal injection of 5 mg/kg Matrine could inhibit the immune rejection after corneal transplantation, which was attributed to increasing the secretion of IL-10 and TGF-β1 in rats. Kan et al. (Kan et al., 2013) showed that Matrine could treat experimental autoimmune encephalomyelitis by inhibiting the migration and infiltration of inflammatory cells into the central nervous system, which was related to significantly downregulate the expression of CVAM-1 and ICAM-1, chemokine CCL3, CCL5, and Toll receptor4 in a dose-dependent manner. Matrine could reduce neurological injury in the mouse model of mild encephalomyelitis, which was related to inhibit the migration of immune cells, prevent the destruction of the blood-brain barrier and prevent the central nervous system from inflammatory infiltration (Jiang et al., 2021).

## Advances on Derivatization of Matrine

Matrine has analgesic effects and the tertiary amino group at the N-1 position is critical for analgesic effects (Wu, 2014; Li, 2015). With modification around N-1 position, Matrine derivatives with lipophilic N-1 structure modification were obtained, and their analgesic activity was enhanced (He et al., 2011). The amide bond may be one of the groups with anti-cancer activity when the lactam of Matrine D-ring was hydrolyzed into Matric acid, the anti-cancer activity disappeared, and after the carboxyl group of Matrine was converted into amide group, the anti-cancer activity recovered. Additionally, the anti-cancer activity was higher than that of Matrine after the conversion of carboxylic acid to esters with longer alkane chain (Hu et al., 2015b). The oxidation or dehydrogenation of Matrine could reduce the toxicity of Matrine, and it was concluded that the toxicity of Sophoramine > Sophocarpine > Matrine (Han, 2012). The molecular conformation of Matrine also affected the anti-tumor activity and Matrine with 5S conformation had better anti-tumor activity than 5R conformation.

Although Matrine has a wide range of pharmacological effects, its low bioavailability and high dosage limit its clinical application. Matrine derivatives were obtained by structural optimization to improve its efficacy and main modification sites were as follows: (1) Tertiary amine at N-1 position. (2) D-ring lactam hydrolysis. (3) C-15 position carbonyl group. (4) C-13 position and C-14 position double bond. (5) C-14 position α-H. The main modification stratigies of Matrine include introducing liposoluble groups and nitrogen-containing heterocyclic structures (N-substituted pyrrole, N-substituted indole, benzenesulfonyl and organic nitrates), combining with active compounds (cisplatin and salicylic acid), and forming complexes with metal ions ([GaCl_4_]^-^ [FCl_4_]^-^ and [AuCl_4_]).

### N-1 Modification

The A and B rings of Matrine were relatively stable with inactive reactivity. However, the tertiary amine at the N-1 position with strong basicity can react with acidic substances to form salts (He et al., 2011). Acetylsalicylic acid, with anti-pyretic and analgesic effects, can react with Matrine to form acetylsalicylic acid Matrine salt (Table 2). It can increase anti-pyretic and analgesic effects and reduce the side effects of acetylsalicylic acid on the intestinal tract and the toxicity of Matrine (Fu et al., 2011). Cinnamic acid has anti-tumor activity. It was combined with Matrine to obtain Matrine cinnamic acid salt to improve its anti-cancer activity (Table 2) (Zhang et al., 2009b). Li et al. (Li, 2015) synthesized a series of derivatives by the reaction of organic nitrates with Matrine, among which 3-nitroxymethylbenzoic acid Matrine (Table 2) had better protective effects on myocardial morphology and structure of isoproterenol-induced myocardial ischemia rats than Matrine. Matrine metal ion complexes (Table 2) exhibited comparable anti-cancer efficacy with combination of Matrine and cisplatin by reaction with [GaCl_4_]^-^ and [AuCl_4_]^-^ (Chen et al., 2011).

**TABLE 2:** 1-position modification of Matrine.

Name of matrine derivative	Basic structure of derivatives	R Substituent structure	Structure of matrine derivatives	Pharmacological activity	Refrences
Matrine acetylsalicylate	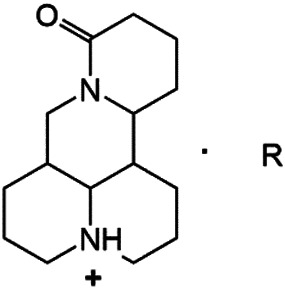	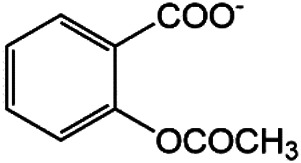	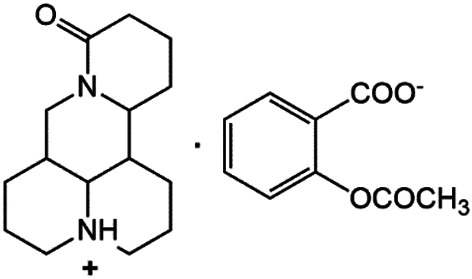	Anti-inflammation and pain relief	Fu et al. (2011)
Matrine 3-nitrooxymethyl benzoate	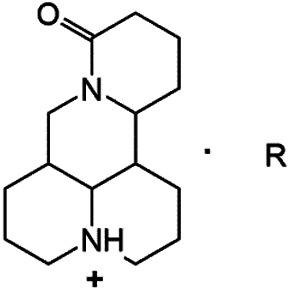	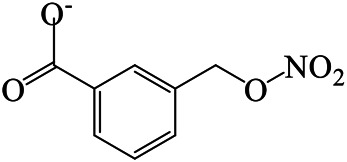	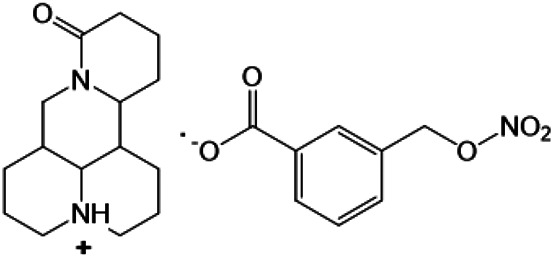	Anti-myocardial ischemia	Li et al. (2015)
Matrine [GaCl_4_] complex	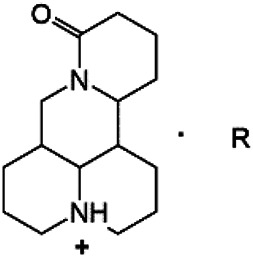	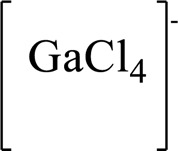	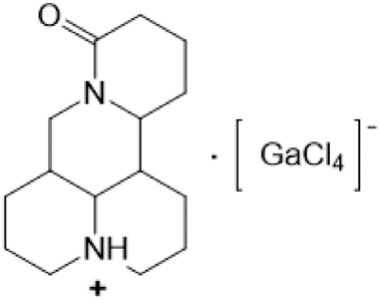	Anti tumor	Chen et al. (2011)
Matrine [AuCl_4_] complex	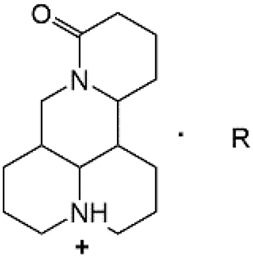	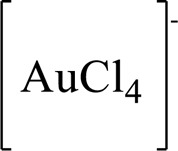	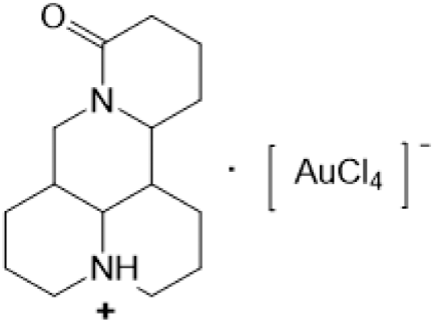	Anti tumor	Chen et al. (2011)

### Hydrolytic Modification of D-Ring Lactam

The D-ring lactam of Matrine was hydrolyzed in an alkaline solution to give stable matric acid with two modified secondary amino and carboxylic groups. The introduction of nitrogen-containing heterocycles is a common strategy for structural modification of natural products, which improves their bioactivity. The bioactivity of Matrine with high soluble in water would be improved by introducing hydrophobic groups (He et al., 2011). Chao et al. (Chao et al., 2013) introduced the benzyl group at the 12-position of Marine and introduced ester, aminoalkyl, and nitrogen-containing heterocycles with different alkyl chain length at the 11-position of Matrine. The new Matrine derivatives exhibited better anti-proliferation activity than Matrine (Table 3). The inhibitory effects of 12-matric acid thiomorphinamide (Table 3) on HepG2 cell proliferation was better than paclitaxel and was 20 times higher than matrine. Amide bond plays a vital role in anti-tumor. Li et al. (Li et al., 2017a) introduced a butanyl side chain at the C-11 position of Matrine and conducted a systematic study on substituting the 12-position atom. It was found that introducing a benzenesulfonyl group at the 12-position gave the derivative 12-N-Benzenesulfonyl kushenbutane (Table 3), which increased the activity against the Coxsackie virus (EC_50_, 2.02 μM for Coxsackie B3, 7.41 μM for Coxsackie A16), which was expected to become a drug candidate for treating Coxsackie virus infection. Matrine was hydrolyzed, matric acid as an intermediate, ethanol was introduced at the C-11 position, and 4-methoxybenzyl was presented at the N-12 position to obtain 12-N-4-methoxybenzyl matrine ethanol (Table 3). It exhibited favorable anti-hepatitis B virus activity (EC_50_, 3.2 μmol) (LI et al., 2020). Fu and TangSLi (Fu and TangSLi, 2014) used 12-benzylmatric acid as the lead compound and replaced the N-12 group with different hydrophobic groups such as n-butyl, n-octyl, n-dodecyl and aromatic groups. The anti-tuberculosis activity and structure-activity relationship of Matrine derivatives were investigated and 12-N-dodecyl *Sophora* flavescens butyl methyl ester (Table 3) showed 16 times high anti-tuberculosis activity than the lead compound. The modification with 11-position ester group, acid group, alcohol group and 12 position hydrophobic group increased anti-tuberculosis effects of Matrine.

**TABLE 3:** Hydrolysis modification of D-ring lactam of Matrine.

Name of matrine derivative	Basic structure of derivatives	R1 substituent structure	R2 substituent structure	Structure of matrine derivatives	Pharmacol-ogical activity	Refrence
Butyl 12-benzyl matrine	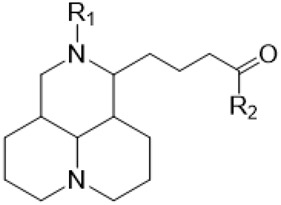	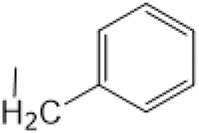	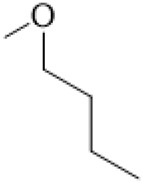	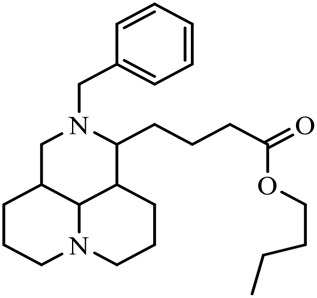	Anti tumor	Chao et al. (2013)
12-matrine thiomorphamide	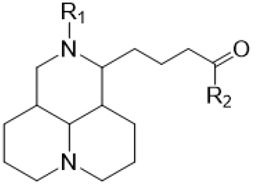	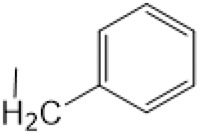	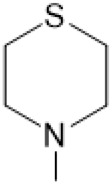	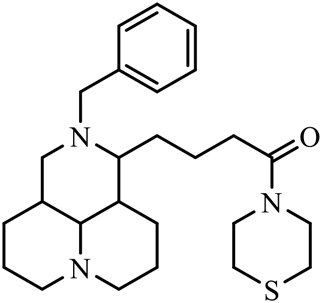	Anti tumor	Chao et al. (2013)
12-N-Benzenesulfonyl Matrine Butane	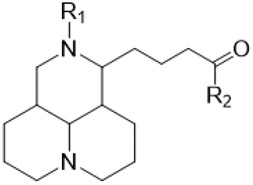	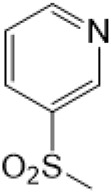	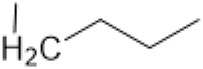	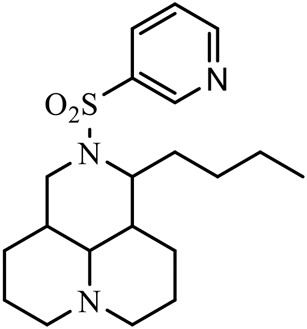	Antivirus	Li et al. (2017a)
12-N-4-Methoxybenzyl Sophora Ethanol	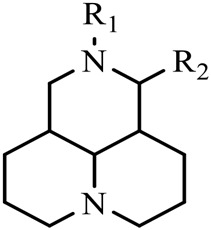	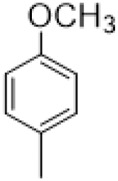	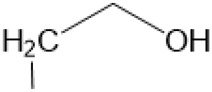	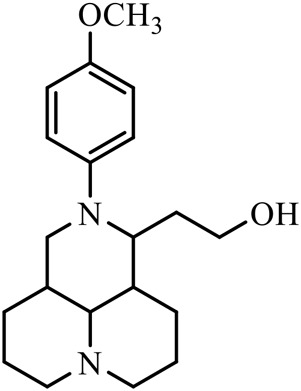	Antivirus	Li et al. (2020)
12-N-dodecyl matrine butyl ester	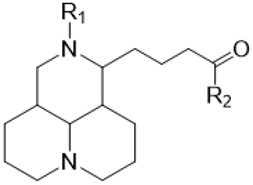	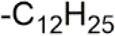	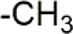	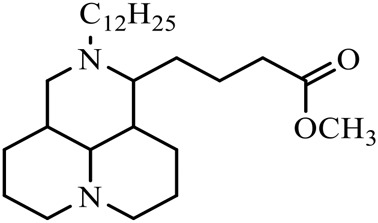	Antivirus	Fu and TangSLi (2014)

### C-15 Modification

There are three main modification strategies at C-15 position of Matrine. (1) Reducing the carbonyl group to an alkyl group by a reducing agent. The lactam of Matrine was reduced to a secondary amine to obtain Deoxymatrine (Table 4), which lost its anti-cancer activity. However, it presented better anti-tobacco mosaic virus activity than Matrine. It was speculated that lone pair electrons enhanced the bioactivity of two nitrogen atoms (Ni et al., 2017). (2) O-atom arrangement of the carbonyl group. The carbonyl oxygen atom at C-15 position of Matrine was replaced with sulfur atom to obtain compound 1 (Table 4), which exhibited comparable bioactivity with Matrine (LI et al., 2020). (3) N-15 position modification. 15-N-substituted Matrine imine derivatives were obtained by synthesizing amidines with aromatic amines and lactams. It was found that (E)-15-(N-4-Biphenyl) Matrine and (E)-15-(N-4-phenoxy phenyl) (Table 4) exhibited favorable *in vitro* anti-tumor activity against human liver cancer cell line HepG2 (IC_50_, <50 μM) and human cervical cancer cell line HeLa (IC_50_, <50 μM) (Hassan et al., 2015).

**TABLE 4:** C-15 position modification of Matrine.

Name of matrine derivative	Name of matrine derivative	Pharmacological activity	Refrence
Deoxymatrine	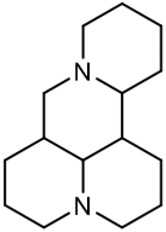	Resistance to tobacco mosaic virus	Ni et al. (2017)
Compound 1	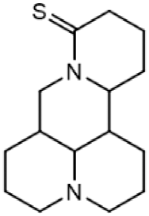	No change in activity	Li et al. (2020)
(E)-15-(N-4-biphenyl) matrine	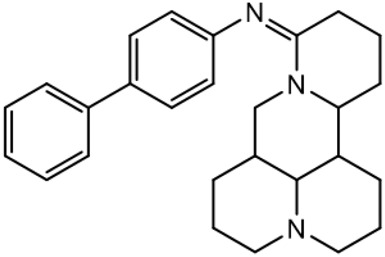	Anti tumor	Hassan et al. (2015)
(E) -15- (N-4-phenoxyphenyl) matrine	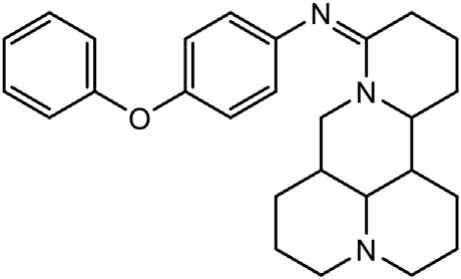	Anti tumor	Hassan et al. (2015)

### C-14 Modification

The αH at 14-position of Matrine was affected by the electron-withdrawing effects of the carbonyl group. The introduction of double bonds and aromatic rings could significantly improve anti-tumor activity. N-substituted pyrrole scaffolds were key groups to increase anti-cancer activity. Zhang et al. (Zhang et al., 2018a) introduced N-(substituted-2-pyrrolylene) and N-(substituted-2-polymethylene) skeletons at C14-position and found that the introduction of benzyl group at nitrogen atom to obtained N-benzyl-2-pyrrolomethyl Matrine (Table 5), which exhibited 115–172 times higher *in vitro* anti-cancer activity against SMMC-7721, A549, and CNE2 cells than Matrine. N-3,5 dimethoxybenzyl-2-pyrromethene Matrine and N-3-chlorobenzyl-3-6 methoxyindole methylene Matrine could significantly promote SMMC-7721 and CNE2 cell apoptosis in a dose-dependent manner. The Matrine derivatives were obtained by aldol condensation reaction with Matrine and 3-methoxy benzaldehyde, 2,3-dimethoxybenzaldehyde, 3, 4, 5-trimethoxybenzaldehyde or 2, 3, 4-trimethoxybenzaldehyde, which exhibited better anti-proliferation activity against cancer cells HT-29 and PANC-1 than Matrine (Wei et al., 2013). The anti-cancer activity of 14-3,4,5-trimethoxybenzylidene Matrine (Table 5) was 3 times higher than Matrine. The number and position of substituted methoxy groups on benzaldehyde affected its anti-cancer activity, and decreasing the steric hindrance could increase the anti-cancer activity. Wu et al. (Wu et al., 2016) introduced an enol group at 14-position of Matrine and replaced the hydroxyl group with the carbonyl group at 15-position to obtain six 6-member ring Matrine derivatives. The optimal compound 2 (Table 5) exhibited 48–109 times higher anti-cancer activity than Matrine. Compound 2 could inhibit cell migration and induce cell arrest at G1 phase of human hepatoma cell lines BEL-7402 and HepG2 by upregulating p21 and p27 and downregulating N-cadherin. Matrine derivatives exhibited favorable anti-proliferative activity against A549, BT-20, MCF-7, and U20S tumor cells by introducing nitrogen heterocycle, oxygen heterocycle, and naphthalene ring into Matrine (Yang, 2015). The optimal derivative 14-[2-(6-Bromo) naphthalene hydroxymethenyl] Matrine (Table 5) exhibited 1000 times higher anti-proliferation activity than Matrine. It could induce A549 cell cycle arrest at G1 phase and promote cell apoptosis by producing ROS in a dose-dependent manner.

**TABLE 5:** C-14 position modification of Matrine.

Name of matrine derivative	Basic structure of derivatives	R Substituent structure	Structure of matrine derivatives	Pharmacological activity	Refrence
N-benzyl-2-pyrromethene matrine	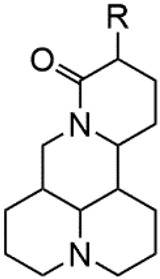	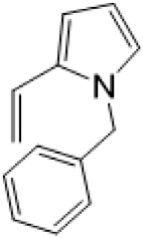	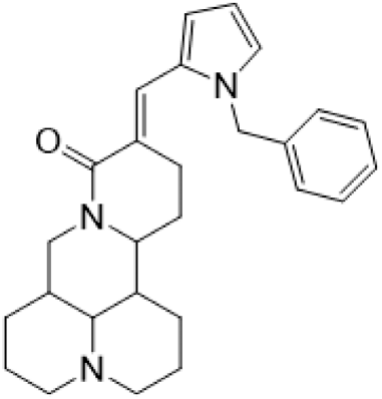	Anti tumor	Zhang et al. (2018a)
N-3,5 dimethoxybenzyl-2-pyrromethene matrine	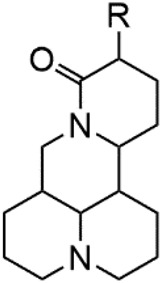	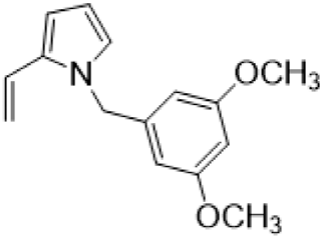	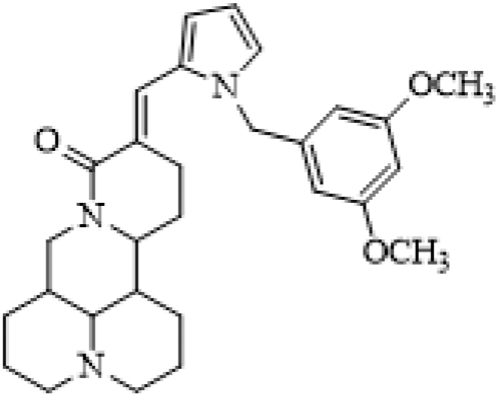	Anti tumor	Zhang et al. (2018a)
N- 3-chlorobenzyl-3-6-methoxyindole methylene matrine	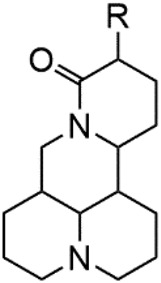	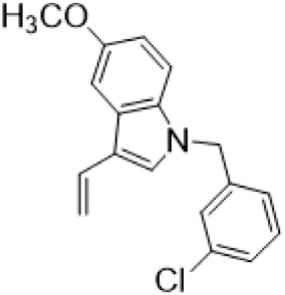	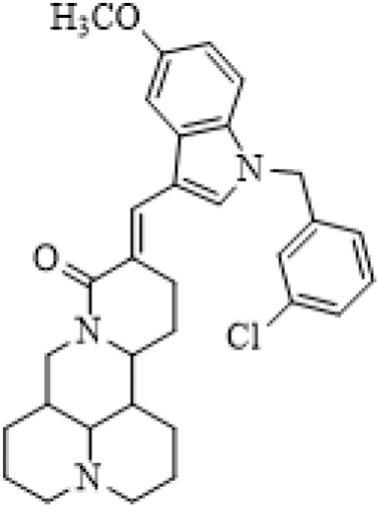	Anti tumor	Zhang et al. (2018a)
14–3,4,5-trimethoxybenzylidene matrine	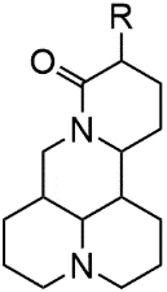	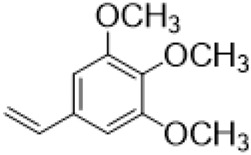	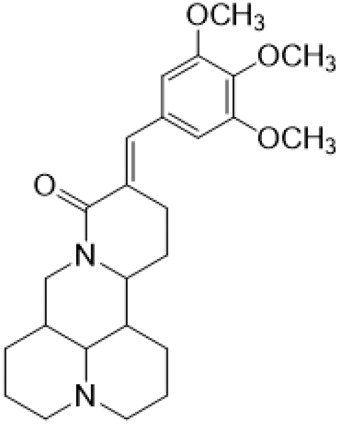	Anti tumor	Wei et al. (2013)
Compound 2	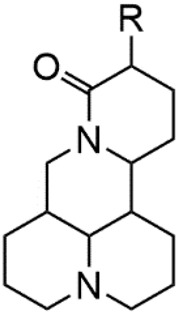	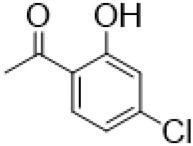	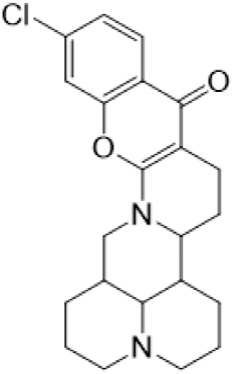	Anti tumor	Wu et al. (2016)
14-[2-(6-bromo) naphthalene hydroxymethenyl] matrine	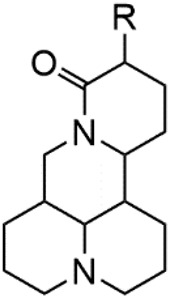	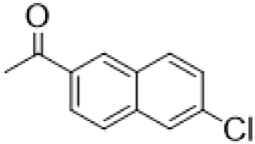	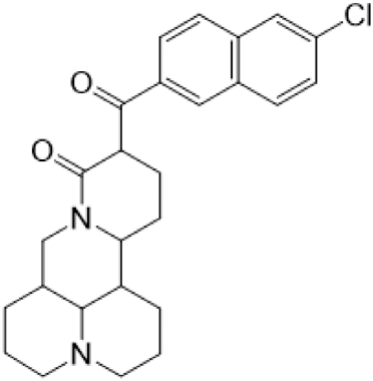	Anti tumor	Yang (2015)

### C-13 Modification

Guo et al. (Guo et al., 2011) replaced the carbonyl group at 15-position with the sulfur atom and introduced different amino side chains at 13-position of Matrine by a classic Michael addition reaction to obtain Matrine derivatives with Sophocarpine as the raw material. The derivative 13-(N-methylene) amino-18-thiomatrine (Table 6) exhibited the optimal anti-cancer activity. The introduction of polyamines can improve anti-inflammation activity of Matrine. Matrine derivative MASM (Table 6), 13-(N-methyl)-amino-18-thiomatrine, exhibited anti-rheumatoid arthritis activity *in vivo* and *in vitro* (Zou, 2018). Han et al. (Han et al., 2012) introduced methoxyl at 13-position to produce 13-α-methoxymatrine with Sophocarpine as raw material, which exhibited better anti-bacterial propagules activity than Matrine. Alkylating agent has always been used as effective chemotherapeutic drug. Cui et al. (Cui et al., 2015) used Sophocarpine as raw material to introduce anti-cancer drugs into Mareine, such as mustard anti-neoplastic drugs melphalan {L3 [p-[bis(2-chloroethyl)amino]phenyl] alanine}, bendamustine (4-[5-[bis(2-chloroethyl)amino]-1-methylbenzimidazol-2-yl] butyric acid) and phosphoryl nitrogen mustard dichloride, the active metabolite of cyclophosphamide compounds 3, 4 and 5 (Table 6). Compound 1 and compound 2 exhibited better anti-tumor activitie than melphalan and bendamustine. Dai et al. (Dai et al., 2019b) synthesized 10 novel 13-hydroxyethyl amine Matrine derivatives. The derivative 13-(N-butyl benzyl) hydroxyethyl amine Matrine (Table 6) exhibited 30 times higher anti-proliferation activity against HepG-2 cells and HeLa cells than Matrine and Sophorine.

**TABLE 6:** C-13 position modification of Matrine.

Name of matrine derivative	R Substituent structure	Structure of matrine derivatives	Pharmacological activity	Refrence
13 - (N-methylene) amino-18 thiomatrine	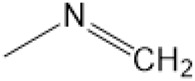	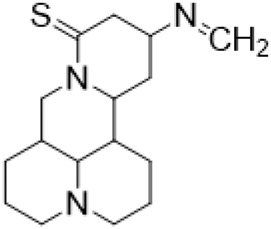	Anti-inflammatory	Guo et al. (2011)
13-(N-methyl)-amino-18-thiomatrine	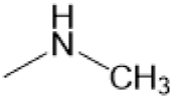	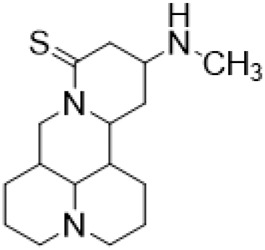	Anti-inflammatory	Zou (2018)
13-α-methoxy matrine	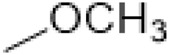	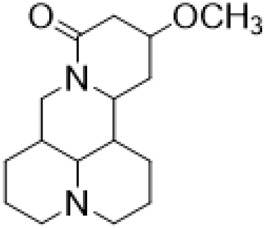	Sterilization	Han et al. (2012)
Compound 3	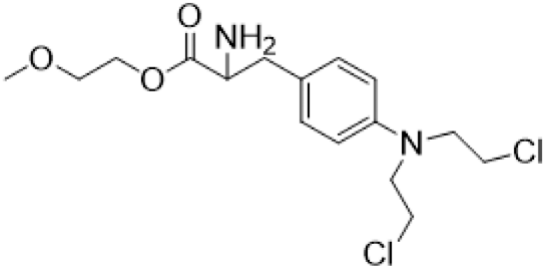	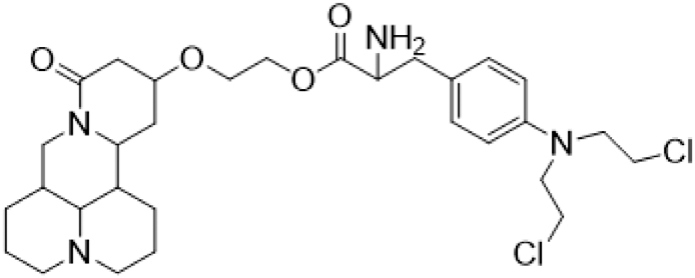	Anti tumor	Cui et al. (2015)
Compound 4	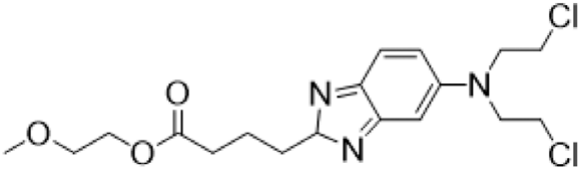	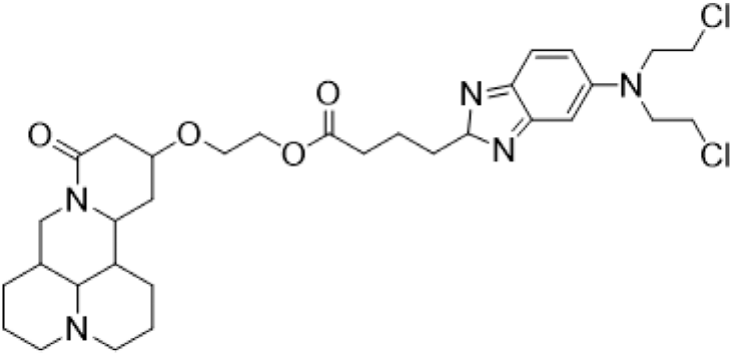	Anti tumor	Cui et al. (2015)
Compound 5	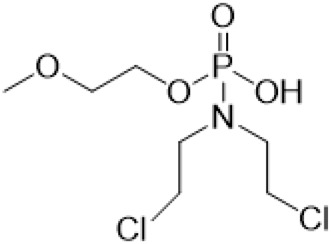	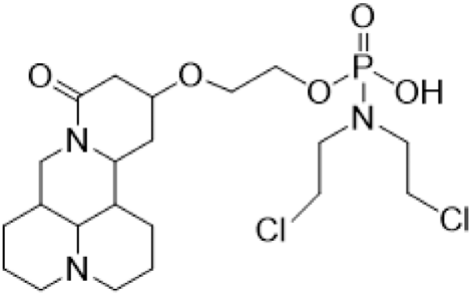	Anti tumor	Cui et al. (2015)
13- (N-4-tert-butylbenzyl) hydroxyethylamin-e matrine	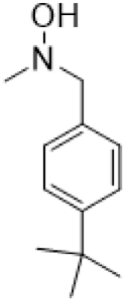	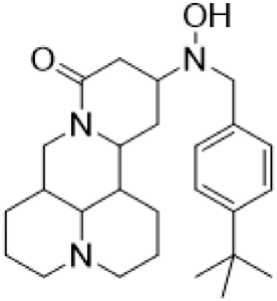	Anti tumor	Dai et al. (2019b)

## Concluding Remarks

Matrine is an alkaloid extracted from traditional Chinese herbs including *Sophora* flavescentis, *Sophora* alopecuroides, *Sophora* root, etc, which was the main resource of Matrine. The common procedures are solvent extraction, ultrasonic aided extraction, and microwave-assisted extraction. Solvent extraction is appropriate for industrial manufacturing, while ultrasonic-assisted and microwave-assisted extraction are suitable for laboratory preparation. But, the yield of Matrine was low by extraction from natural plants. Researchers developed three main total synthesis strategies to increase the yield of Matrine. However, most of the synthesis routes were long, the synthesis conditions were not easy to control, and the yield was low (He et al., 2011), which needed further study to develop optimal synthesis strategies.

Matrine has the dual advantages of traditional Chinese herbs and chemotherapy drugs and exhibits distinct benefits in preventing and improving chronic diseases such as cardiovascular disease and tumors. Matrine exhibited favorable activity on anti-atherosclerosis, anti-hypertension, anti-ischemia reperfusion injury, anti-arrhythmia, anti-diabetic cardiovascular complications, anti-tumor, anti-inflammatory, anti-bacterium, anti-virus. But the molecular function mechanism of Matrine is in the air, which needs further research works to explore.

Although Matrine exhibited multiple bioactivities, its low bioavailability and high dosage limit its clinical application. Matrine derivatives were obtained by structural optimization to improve its efficacy and main modification sites were as follows: (1) Tertiary amine at N-1 position. (2) D-ring lactam hydrolysis. (3) C-15 position carbonyl group. (4) C-13 position and C-14 position double bond. (5) C-14 position α-H. The main modification stratigies of Matrine include introducing liposoluble groups and nitrogen-containing heterocyclic structures (N-substituted pyrrole, N-substituted indole, benzenesulfonyl and organic nitrates), combining with active compounds (cisplatin and salicylic acid), and forming complexes with metal ions ([GaCl_4_]^-^ [FCl_4_]^-^ and [AuCl_4_]). Various Matrine derivatives exhibited better bioactivity than Matrine, which would provide new core structures and new insights for new drug development in related fields. There are still further research works needed to be done to obtained optimal derivatives with high bioactivity and low side effects. With the further research in derivatization and molecular mechanism, Matrine would have wider application prospects in the future.
